# Bacterial Communities Associated with Houseflies (*Musca domestica* L.) Inhabiting Hospices in South Africa

**DOI:** 10.3390/microorganisms11061440

**Published:** 2023-05-30

**Authors:** Maropeng C. Monyama, Oriel M. Taioe, Jane S. Nkhebenyane, Deidre van Wyk, Tsepo Ramatla, Oriel M. M. Thekisoe

**Affiliations:** 1 Department of Life and Consumer Sciences, University of South Africa, Florida 1710, South Africa; 2 Unit for Environmental Sciences and Management, North-West University, Potchefstroom 2520, South Africa; taioem@arc.agric.za (O.M.T.); 20418876@nwu.ac.za (D.v.W.); ra21205450@gmail.com (T.R.); Oriel.Thekisoe@nwu.ac.za (O.M.M.T.); 3 Epidemiology, Parasites and Vectors, Agricultural Research Council-Onderstepoort Veterinary Research, Pretoria 0110, South Africa; 4 Department of Life Sciences, Central University of Technology, Bloemfontein 9300, South Africa; snkheben@cut.ac.za

**Keywords:** bacterial communities, *16S rRNA*, houseflies, resistance genes

## Abstract

Houseflies are alleged reservoirs as well as vectors of human and animal pathogens, including bacteria, because they frequently have contact with animal excreta and decaying organic substances. The rapid adaptation process of ingested microbes in the insect gut may involve gene transfer, including antibiotic resistance determinants among different bacterial strains. Six hundred and fifty-seven (n = 657) houseflies were collected from hospices and were identified morphologically and genetically using the *16S rRNA*, *CO1*, and *ITS2* barcoding genes. This study also characterized the bacterial communities harboured by the captured houseflies using 16S rRNA metabarcoding on the next-generation sequencing (NGS) platform and further sought to detect antibiotic resistance traits by using gene-specific PCR assays. Generated sequences for the targeted gene fragments matched with *Musca domestica* and all the sequences were deposited to the GenBank database. The 16S rRNA metabarcoding analysis revealed that the most abundant phyla detected with variable abundance observed among all the houseflies were *Proteobacteria*, followed by *Firmicutes*, and *Bacteroidetes*. Furthermore, the NGS data revealed the presence of multiple bacterial genera, including *Providencia*, *Enterobacter*, *Dysgonomonas*, *Escherichia-Shigella*, *Klebsiella*, *Pseudomonas*, and *Streptococcus*, which are known to harbour potentially pathogenic species of animals and humans. Antibiotic resistance genes detected from the housefly DNA in this study included *ermB*, *tetA*, *blaSHV*, and *blaTEM*. Moreover, these genes are associated with resistance to erythromycin, tetracycline, and beta-lactams antibiotics, respectively. The presence of bacterial pathogens and the detection of antibiotic resistance genes from the houseflies collected from the hospices indicates the possible health risk to patients in hospices and the surrounding community. Therefore, it is imperative to keep high standards of hygiene, food preparation, safety, and control of houseflies in hospices.

## 1. Introduction

The association of bacterial communities and muscoid flies, particularly *Musca domestica*, has been studied from numerous viewpoints, including: (i) the importance of microbes for larval development, (ii) the digestibility of bacteria in the intestinal tract of housefly larvae, and (iii) the potential transmission of bacterial pathogens by adult flies [1,2]. As such, these flies tend to be associated with microbe-rich habitats, including domestic and agricultural settings, for reproductive purposes [3]. These settings are mainly packed with diverse and active microbial communities, creating an ideal opportunity for houseflies to gather and potentially transmit pathogenic organisms to human and animal food [4,5].

Houseflies are highly mobile and theoretically transport bacterial cells from highly contaminated substrates from various settings [6]. Thus, this insect can transmit pathogenic microorganisms by contaminating different parts of their external body surface, including their feet, wings, appendages, and mouthparts [3,4,5]. The housefly’s exoskeleton on the aforementioned organs enables easy transmission of antibiotic-resistant pathogens and medically relevant bacteria to surfaces [7].

Houseflies colonized with bacterial species could also be associated with the spreading of antibiotic resistance genes or exposure of virulent bacterial strains within the same setting [8,9]. Thus, the risk of houseflies in distributing resistant bacterial strains from domestic and agricultural settings to places including hospices is of great public health concern. In the South African context, hospices are places that house terminally ill patients suffering from diseases such as cancer and are also used to care for acquired immune deficiency syndrome (AIDS) patients [10,11]. Therefore, as non-governmental organizations, hospices provide active total care for patients whose disease is non-reactive to treatment and as such their goal is to attain a good quality of life for patients and their families [12].

The microbiological culture method is one of the most broadly used tools for microbial organisms’ identification and is considered as a ‘gold standard’ because of its ability to detect new bacterial species and test their susceptibility or resistance to antibiotics [13]. However, many bacteria are uncultivable, especially those harbored by insects [14]. They have been reported to be difficult to isolate, or they grow slowly in the culture due to stringent growth requirements or may not grow because of prior empirical treatment with antimicrobial agents [15,16]. Thus, high-throughput DNA sequencing methods bring innovative opportunities to characterize bacterial communities [16]. This allows identification of both cultivable and uncultivable bacteria in an effort to accomplish an extended perspective on bacterial diversity with higher coverage and a focus on a different set of organisms [16].

To detect antibiotic-resistant pathogens and their resistance genes, the polymerase chain reaction (PCR) technique, with the aid of species-specific oligonucleotide primers and probes, have been developed [17]. Recently, the use of PCR to detect the presence of antibiotic resistance genes in a bacterial isolate as well as in samples from different environments has been made familiar [18]. Hence, the present study characterized the bacterial communities harboured by houseflies collected from hospices using next-generation sequencing on the Illumina MiSeq platform and aimed to detect antibiotic resistance genes by gene-specific polymerase chain reaction assays.

## 2. Materials and Methods

### 2.1. Housefly Sampling

Houseflies were collected from one hospice (Sparrow) in Johannesburg and two hospices (Lebone and Sunflower) in Bloemfontein between September 2019 and February 2020, respectively. In all sampled hospices, a minimum of three sticky traps were set for at least three to five days for a period of two weeks in the kitchens and dining halls to capture houseflies. The sticky traps were monitored daily. Houseflies were immediately stored in a properly labeled container with 70% ethanol and transported in an icebox to the laboratory for processing. PCR assays targeting the 16*S rRNA*, *CO1*, and *ITS*2 genes were conducted to identify houseflies to the species level as well as to supplement morphological observations. Twenty microliters of all positively amplified PCR products were sent to a commercial sequencing facility, at Inqaba Biotechnical Industries (Pty) Ltd., Pretoria, South Africa for purification and sequencing in both directions.

### 2.2. DNA Extraction from Houseflies

Prior to DNA extraction, housefly specimens were washed twice with 70% ethanol and once with double distilled water (ddH2O) to remove any contaminants from the environment. Subsequently, captured flies were pooled according to their sites of collection based on the day of the sampling, i.e., five flies per pool. Moreover, the pooled samples were crushed into sterile 1.5 mL Eppendorf tubes and subjected to DNA extraction using Qiagen DNeasy PowerSoil Kit (QIAGEN, Hilden, Germany) according to manufacturer’s instructions. Ultimately, genomic DNA was eluted with 100 μL of solution C6 from the kit. The extraction yield and DNA quality were verified by 1% agarose gel electrophoresis.

The quantity of DNA extracted from the samples was determined by spectrophotometry with a NanoDrop ND-1000 system (NanoDrop Technologies, Inc., Wilmington, DE, USA). The purity of DNA was determined spectrophotometrically from the ratio of absorbance at 260 and 280 nm (A260/A280). A ratio of between 1.7 and 2 indicates an excellent quality of DNA [19].

### 2.3. Partial 16S rRNA Gene Amplicon Sequencing

Total genomic DNA was amplified as recommended by Illumina MiSeq 16S Metagenomic Sequencing Library Preparation Guide. The libraries of partial 16S rRNA gene (hypervariable V3–V4) were amplified using published universal bacterial primers by Klindworth and colleagues [20].

Briefly, the library preparation protocol entailed a first “amplicon PCR” step involving 12.5 ng DNA, 0.2 µM of each forward and reverse primers, 12.5 µL of KAPA HiFi HotStart ready Mix (0.5 U DNA polymerase, 0.3 mM dNTPs, 2.5 mM MgCl2) (Kapa Biosystems, Wilmington, MA, USA) and nuclease-free water (Thermo Fisher Scientific, Waltham, MA, USA) in a final reaction volume of 25 µL. Furthermore, PCR amplicons were purified with Agencourt AMPure XP magnetic beads (Beckman Coulter, Brea, CA, USA). The libraries were amplified with a limited-cycle PCR program (10 cycles) to add the index 1 (i7) and index 2 (i5) adapters, containing sequences required for cluster generation of the Illumina flow cell. The quality and sizes of the resulting DNA fragments were evaluated on a 2% (*w*/*v*) agarose gel. The libraries were quantified with a fluorometric method (Qubit, Life Technologies) and normalized to 4 nM using a standard dilution method. The libraries were pooled, denatured with 0.1 N NaOH, and diluted to the final loading concentration of 6 pMol. An identically treated PhiX Control v3 adapter-ligated library at a low-concentration spike-in of 10% was added as an in-lane positive control for alignment calculations and quantification efficiency. Paired-end sequencing was carried out on an Illumina MiSeq system (Illumina, San Diego, CA, USA) using a MiSeq Reagent Kit V3 600 cycles.

### 2.4. Metagenomic Data Analysis

#### Bioinformatic and Diversity Analyses

Demultiplexed paired-end reads were checked for quality using FastQC software (version 0.11.5, Babraham Institute, United Kingdom). Reads were then denoised (i.e., further quality filtering, error correction, and removal of chimeric sequences) and clustered into amplicon sequence variants (ASVs) by using the DADA2 denoiser [21] integrated into the Quantitative Insight into Microbial Ecology version 2 (QIIME2) software [22]. The denoised reads were assigned taxonomy by the Silva *16S rRNA* taxonomy (Release 132) [23] using a trained classifier of the V3–V4 region. After that, singletons and non-bacterial taxa (e.g., Archaea, mitochondria, and chloroplast) were eliminated. Alpha diversity indices, including the number of observed ASVs (ASV richness, R), Chao1 richness estimation, the Shannon–Wiener index (H’), and the species dominance (D) were computed in QIIME 2 software. Downstream statistical analyses were carried out using Microbiome Analyst [24,25] online web tool with Marker Data Profiling (MDP) module. A total of 1,720,766 ASVs were recovered after removing the singletons. Further filtering with default parameters yielded 273 ASVs. The normalization was carried out to rarefy the data to the minimum library size (14,282) using the total sum scaling option. Alpha diversity was calculated using Observed, Chao1, Shannon, and Simpson diversity measures. Community ASV comparisons were visualized by Principal coordinates analysis (PCoA) using the Bray–Curtis dissimilarity index and the differences were quantified using permutational multivariate analysis of variance (PERMANOVA). Metacoder software [26] was implemented in R (R Core Team 2020) (Version., 3.6.1) for the construction and visualization of heat tree using the ASVs and taxonomy file from QIIME 2.

### 2.5. Screening for Antibiotic Resistance Genes

Antibiotic resistance genes were selected based on their clinical and medical importance [27] and because they are of common concern in environmental samples [28]. The presence of nine antibiotic resistance genes including β-lactam (*blaCARB*, *blaTEM*, and *blaSHV*) tetracycline (*tetA*, *tetW*, and *tetX)*, and macrolide (*ermB*, *mecA*, and *vanA*) were detected by polymerase chain reaction (PCR) assays. Thus, the primers used to determine the presence or absence of the above-mentioned antibiotic resistance genes and class 1 and 2 integrons in housefly DNA samples, were synthesized at Inqaba Biotechnical Industries (Pty) Ltd., Pretoria, South Africa based on the published literature, with the details listed and described in Table 1.

### 2.6. PCR for Amplification of Resistance Genes

All PCR reactions were performed with the final reaction volume of 25 µL, which consisted of 1 μL of template DNA (±100 ng/μL), 8.5 μL of ddH_2_O, 12.5 μL of 2X PCR master mix with standard buffer (OneTaq Quick load buffer, 4 mM MgCl_2_, 0.4 mM of each dNTP and 1 unit/μL of thermostable Taq polymerase) (Thermo Scientific, USA); the primer mix contained 1 μM of each oligonucleotide primer.

The PCR reactions were performed as follows: initial denaturation at 94 °C for 6 min, followed by 30 cycles of 94 °C for 30 s, annealing temperature (Table S1) for 30 s, and 72 °C for 60 s, with a final extension at 72 °C for 6 min. The presence of resistance and virulence genes was determined by the PCR amplicon band of the expected size in 1% agarose gel electrophoresis in 1× TAE buffer run at 100 V for 30 min. Both negative control (pure sterile water) and positive control (16*S rRNA*) (Table S1), according to [28] were included in the PCR run to ensure the correct operation of PCR reactions.

## 3. Results

### 3.1. Housefly Collection

Six hundred and fifty-seven (N = 657) houseflies were collected from the three sampled hospices, whereby 306 (46.6%) were from Lebone hospice, 258 (39.3%) from Sparrow hospice and 93 (14.2%) were from Sunflower hospice. The houseflies were morphologically identified as *Musca domestica* and further confirmed by PCR and sequencing of three genes which matched with the relevant species on the NCBI GenBank database. The sequences generated in this study were submitted on the GenBank database with the following accession numbers: MZ702642; MZ702643 and MZ702644 for *16S rRNA*, MW579487; MW579488; MW579489; MW579490; MW579491 and MW579492 for *CO*1 and MW584801; MW584802; MW584803; MW584804; MW584805; MW584806; MW584807; MW584808; MW584809 and MW584810 for *ITS*2. A total of 44 samples were used for *16S rRNA* metagenomics amplification, whereby 14 samples were from Lebone hospice, 18 samples from Sparrow hospice and 12 samples were from Sunflower hospice. A total of 45 samples, i.e., fifteen samples from each hospice, were used to detect antimicrobial resistance.

### 3.2. Summary of Generated ASV

A total of 1,720,766 reads were obtained with an average count of 39,108 reads per sample. Using the Microbiome Analyst for data analysis following the filtration step, 589 low abundance features and 14 low variance features were removed based on the interquartile range. The remaining features after the data filtering step were 121, and subsequently, a total of 4871 ASVs were generated from this study. The produced ASVs were then classified into phyla, classes, orders, families, and genera.

### 3.3. Taxonomic Classification of Bacteria

The detected microbiota composition at the phylum level from the three sampled hospices showed three dominant phyla. The most abundant phyla detected with variable abundance observed among all houseflies were Proteobacteria, followed by Firmicutes, and Bacteroidetes (Figure S1). The most abundant class detected from the three hospices were Gammaproteobacteria, Bacilli, Bacteriodia, and Alphaproteobacteria (Figure S2). However, Alphaproteobacteria was observed in some of the samples from the Lebone and Sparrow hospices and only in one sample from the Sunflower hospice.

Enterobacteriaceae was the most dominant bacterial family observed in all the houseflies of all the sampled hospices, followed by other families including, Enterococcaceae, Dysgonomonadaceae, Weeksellaceae, Cardiobacteriaceae, Streptococcaceae, Rhizobiaceae, and Leuconstocaceae to list a few (Figure S3).

**Figure 1:**
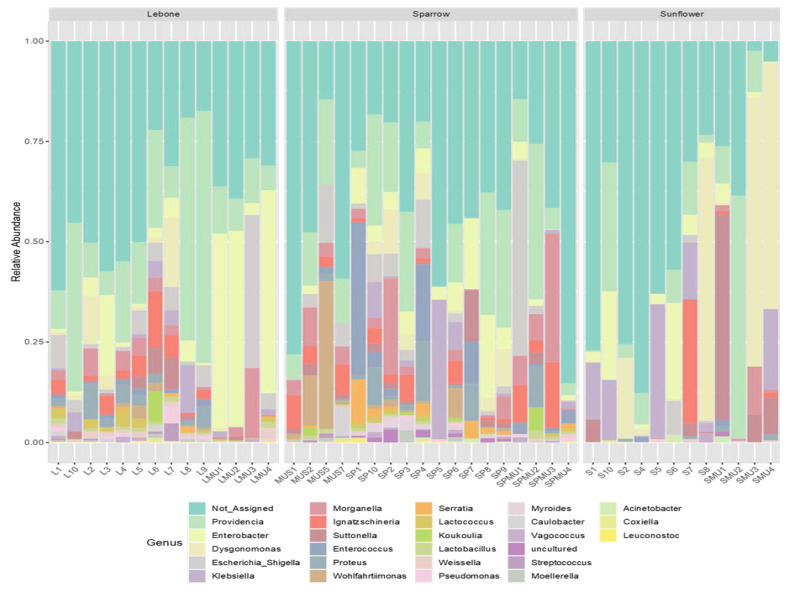
A stacked bar plot showing relative abundance of bacterial genera detected from houseflies collected from three hospices in Bloemfontein and Johannesburg cities. In the x-axis, L and LMU represents samples from Lebone hospice; SP and SPMU represents samples from Sparrow hospice; whilst S and SMU represents samples from Sunflower hospice.

The microbiota composition associated with the houseflies at the genus level were assessed whereby *Providencia* was the most abundant bacterial genera from all the analyzed samples. This was followed by *Enterobacter*, *Dysgonomonas*, *Escherichia-Shigella*, *Klebsiella*, and *Morganella* (Figure 1). Additionally, other genera included *Ignatzschineria*, *Subtonella*, *Enterococcus*, *Proteus*, *Wohlfahotimonas*, as well as *Enterococcus*, *Serratia*, *Streptococcus*, *Pseudomonas*, and *Coxiella* that were also detected across the tested housefly samples.

### 3.4. Alpha Diversity Index

**Figure 2:**
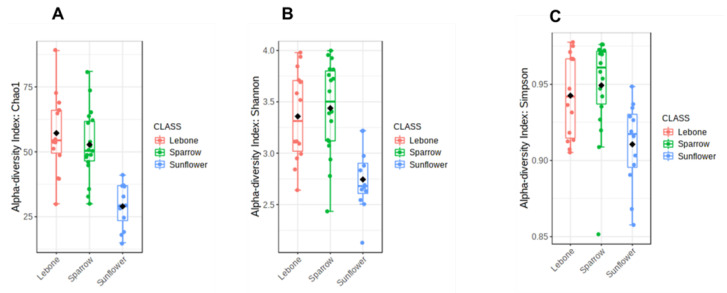
Boxplots showing the alpha diversity comparisons of housefly samples from hospices. (**A**): Chao1 index; (**B**): Shannon index; (**C**): Simpson index.

Alpha diversity was used to evaluate the diversity differences at the ASVs level using Chao1, Shannon, and Simpson indices, respectively. The observations from Chao1 index showed the variation in species abundance and richness of housefly bacterial communities from individual samples (Figure 2A). The samples from Lebone and Sparrow hospices were significantly more diverse. The samples from Sunflower hospice showed low microbial communities as compared to the other hospice samples. The Shannon alpha diversity index (Figure 2B) showed no significant variation among the housefly samples obtained from the Lebone and Sparrow hospices. The high bacterial diversity observed from the Lebone and Sparrow hospices was supported by a high Simpson index (Figure 2C) where almost all three hospices showed an average Simpson index of more than 0.9; thus, the houseflies presented a diverse habitat regarding the microbiome.

### 3.5. Beta Diversity Index

**Figure 3:**
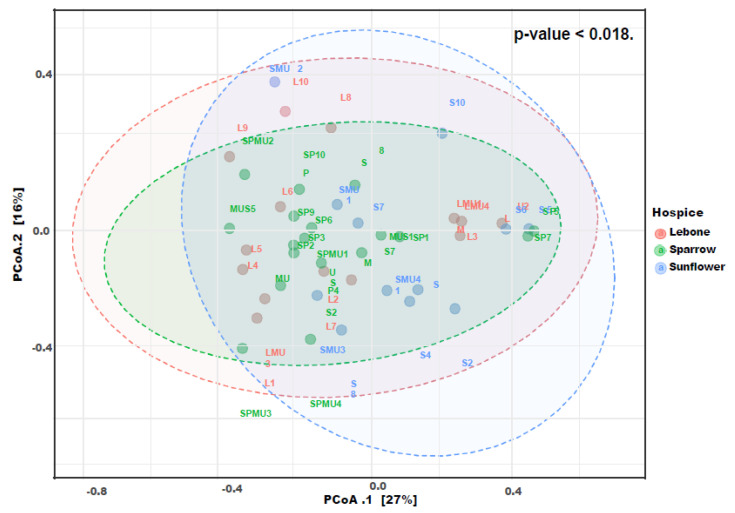
Multivariate differentiation of bacterial communities in hospices statuses. Dotted lines show the distance of every sample to its group centroids in multivariate space, while ellipses show 95% confidence intervals (standard error) in multivariate space around group centroids. The PERMANOVA *p*-values are also indicated in this figure.

**Figure 4:**
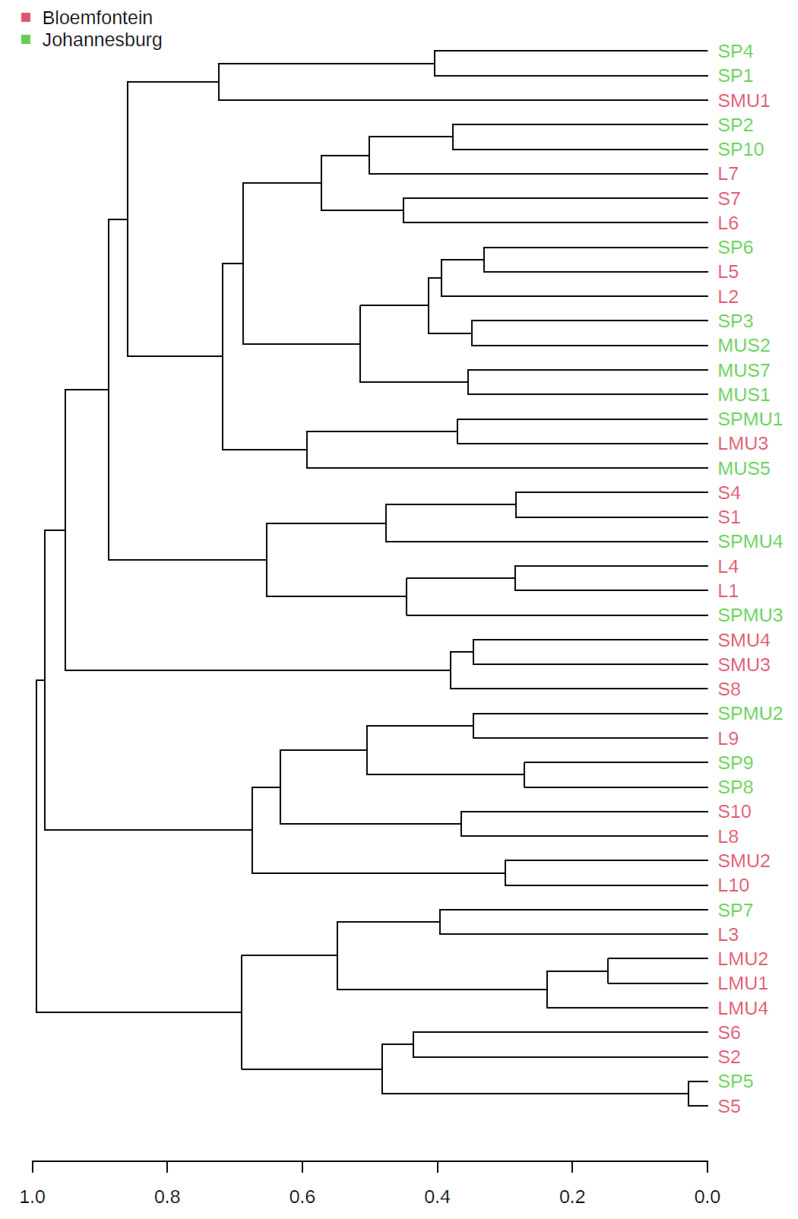
Cluster dendrogram showing how the ASVs from various locations clusters. Clustering was by location. The Bray–Curtis index measure and ward clustering method was used to generate the dendrogram.

Community ASV comparisons were visualized by Principal coordinates analysis (PCoA) and cluster dendrogram based on the Bray–Curtis dissimilarity index (Figure 3 and Figure 4). The Bray–Curtis index is used to quantify the compositional dissimilarity between two different sites, based on the counts per site. As such, our results showed no apparent clustering between the housefly samples obtained from the three hospices. This observation revealed that the housefly samples shared some bacterial species, if not all. The observed differences in a multivariate space among the hospices in Bloemfontein and Johannesburg were significant (PERMANOVA *p* < 0.05; Figure 3). A dendrogram (Figure 4) showed that ASVs clustered by location.

### 3.6. Antibiotic Resistance Genes

Nine (9) targeted antibiotic resistance genes and two integrons (Table 1) were selected for this study and detected by conventional PCR. Accordingly, only 44.4% (4/9) of the antibiotic resistance genes, including *ermB*, *tetA*, *blaSHV*, *and blaTEM* were detected in the housefly samples from the hospices as summarized in Table 1. Whilst 55.6% (5/9) of the antibiotic resistance genes including *blaCARB*, *vanA*, *tetX*, *tetW*, and *mecA* were not detected.

### 3.7. Occurrence of Antibiotic Resistance Genes

#### 3.7.1. β-Lactam Resistance Gene

The *blaTEM* and *blaSHV* were the only detected β-lactam resistance genes. The *blaTEM* gene was present in 26.7% of the samples from Lebone hospice and 13.3 % from the Sunflower hospice samples. No *blaCARB* and *ampC* were detected from the tested housefly samples. Additionally, no β-lactam antibiotic resistance genes were detected from samples obtained from Sparrow hospice.

#### 3.7.2. Macrolide Resistance Genes

The *ermB* was the only penicillin resistance gene detected from both Lebone hospice and Sparrow hospice with the detection frequency of 13.3% and 6.6%, respectively. Neither of the *mecA* and *vanA* genes were detected in the samples obtained from all three sampled hospices. No macrolide antibiotic resistance gene was detected from the samples obtained from Sunflower hospice.

#### 3.7.3. Tetracycline Resistance Genes

The *tetA* gene was detected from the housefly DNA samples obtained from all the sampled hospices with the detection frequency of 60%, 33%, and 20% from Lebone, Sunflower, and Sparrow hospices, respectively. Additionally, no *tetX* and *tetW* resistance genes were detected from all the tested housefly samples.

#### 3.7.4. Integrons

Class1 and class 2 integrons genes were detected in the housefly DNA extract samples obtained from all the sampled hospices. The detection frequency of Class1 integrons from Lebone, Sparrow, and Sunflower hospices was 53.3%, 40.0%, and 46.7%, respectively. The detection frequency of Class 2 integrons from Lebone, Sparrow, and Sunflower hospices was 60.0%, 13.3%, and 53.3% respectively.

## 4. Discussion

This study explored the bacterial communities and the occurrence of antibiotic resistance genes associated with houseflies (*M. domestica*) from the hospices in two of the major cities of South Africa. The results provided a culture-independent description of the microbiota of a typical house fly (*M. domestica*), a vector of public health importance. The abundant phyla detected in this study included Proteobacteria, Firmicutes, and Bacteroidetes. Similar results were obtained by [29], where they assessed the houseflies sampled within and between farms (town/city and country). Additionally, a study by Zhao et al. [16] indicated that these bacterial phyla were present and dominating in the larval stage of houseflies. However, de Jonge et al. [30] revealed that the changes in the microbial community composition through the different housefly developmental stages were characterized by a diversity of microbes colonizing the larval stage, followed by an exchange of new microbiota. The change observed was of highly abundant colonization by Bacteroidetes on adult flies to Firmicutes during the developmental stages and a combination of Bacteroidetes, Firmicutes, and Proteobacteria from the adult housefly samples. Microbial phyla observed in this study have been found to be associated with several other species of arthropods, including *Manduca sexta* (Tobacco hornworm), *Helicoverpa armigera* (Cotton bollworm), *Aedes albopictu*s (Asian tiger mosquito), *Apis mellifera* (Western honeybee), *Culex quinquefasciatus* (Southern house mosquito), flesh fly (*Sarcophaga* sp.), and blowflies (Calliphoridae) [5,31,32,33,34,35,36,37]. In this study, bacteria were identified to the genus level. The prevalent genera identified in this study included *Providencia*, *Enterobacter*, *Dysgonomonas, Klebsiella*, *Escherichia-Shigella*, *Pseudomonas*, and *Staphylococcus*, which are known to harbour potentially pathogenic species of clinical and veterinary relevance. These findings are in accordance with the systematic review by Khamesipour et al. [38] who observed the dominant pathogenic bacteria in houseflies captured from different environments. The study by Park et al. [39] observed the genera *Streptococcus* and *Escherichia-Shigella* to be more prevalent in housefly samples collected from hospitals and farms, respectively.

The *Providencia*, the most dominant genera detected, is a genus of ubiquitous Gram-negative bacteria in the family Enterobacteriaceae and causes several human diseases [5,16]. Members of the genus *Providencia* have been isolated from a range of environmental niches and living organisms. *Providencia* is comprised of other opportunistic pathogens of humans and insects [39]. However, this genus has been identified as part of the normal human gut flora [16]. However, *Providencia* has been reported to be beneficial in carrion-feeding insects, including blowflies [40]. These genera were found to have nutritional benefits by producing several xylanases that help in the decomposition of xylan observed at decomposition sites [40].

Firmicutes were also a major component in the gut of the houseflies obtained in this study. The genus *Staphylococcus* was the second most abundant genera detected from the housefly samples in this study. Similar observations were made by the other studies on houseflies studied in the United States [41], India [5], and again in the United States [1]. The genera *Streptococcus* and *Micrococcus* were more prevalent in the hospital fly samples, whereas *Clostridium* and *Escherichia-Shigella* were more prevalent in the farm fly samples [39]. However, no *Clostridium* and *Micrococcus* genera were detected in the current study. There was a presence of harmful genera, including *Coxiella*, in some of the housefly samples. This is concerning since this genus causes Q-fever, which may present as an acute febrile illness with pneumonia or as a chronic infection with endocarditis [42,43].

The occurrence of antibiotic resistance genes in housefly microbial communities from three hospices revealed the highest prevalence of antibiotic resistance genes from Bloemfontein in Lebone hospice, followed by Sunflower hospice, with Sparrow hospice based in Johannesburg showing the least prevalence. These occurrences might be due to the agricultural activities, such as the nursery, which produces seedlings for vegetable production, broiler chicks, and piggeries around the Lebone hospice area and Sunflower hospice being situated next to the hospital. This type of agricultural activity involves using antibiotics as prophylactics to prevent and limit the spread of diseases and as growth promoters in production [44]. Thus, antibiotics are widely used in animal husbandry, and various types of antibiotic resistance genes are frequently detected in livestock waste around the world [44]. The presence of antibiotics in the environment may provide long-term selective pressure for the emergence and transmission of these resistance-conferring bacteria [44,45]. Although there was a notable numeric difference amongst the detected resistance genes in this study, there was no statistical significance observed among the classes of antibiotic resistance genes.

The *blaSHV* gene detection predominated *blaTEM* genes detection in the samples from Lebone and Sunflower hospices. The gene *blaTEM* was also detected from *Pseudomonas aeruginosa* isolated from housefly samples in Iran [46]. The results are in accordance with a study by Eftekhar et al. [47] on the detection of extended spectrum β-lactamase in urinary isolates of *Klebsiella pneumoniae*, in which *blaSHV* exceeded *blaTEM*; however, they are in disagreement with the findings by Yazdi et al. [48] who reported that the most prevalent β-lactamase-encoding gene was *blaTEM* followed by *blaSHV*. Thus, their increasing prevalence and shocking evolution seem to be directly linked to their clinical use [49,50].

The *ermB* gene was the only detected macrolide resistance gene from the housefly microbial communities captured in Lebone and Sparrow hospices from this study. This gene is recognized as one of the four classes of erm resistance determinants that are correlated with disease-causing microorganisms [51]. Detecting this resistance gene in housefly microbial communities signifies the presence of resistant bacteria against macrolide. Detection from housefly microbial communities is not surprising because this gene has been detected in agricultural settings and primary animal production facilities [52]. Thus, houseflies can easily be contaminated by antibiotic-resistant bacteria due to their indiscriminatory movements.

The tetracycline resistance genes found at the highest frequency in Gram-negative bacteria are related to efflux pumps, which are coded by the *tetA, tetB, tetC, tetD,* and *tetG* genes [53]. However, the occurrence of tetracycline resistance genes encoding ribosomal protection proteins was examined in the housefly microbial communities from hospices in this study. Thus, the tetracycline efflux protein-encoding gene *tetA* was detected from all the sampled hospices, with Lebone hospice expressing high detection frequencies. In a study by Akter et al. [54], five antibiotic resistance genes were detected from three organisms isolated from houseflies, where *tetA* was the most common resistance gene isolated. Notably, *tetA* genes have been associated with anthropogenic impacts occurring in the surrounding, and a number of these genes have been found in various pollution sources. The presence of the *tetA* gene in all the samples collected from all the sampled hospices could be credited to its high abundance in the environment [55]. This efflux protein-encoding gene *tetA* has previously been shown to be present in both hospitals and aquatic environments [54].

Integrons are believed to play a major role in rapidly disseminating multi-drug resistance among bacteria [56]. The results obtained in this study revealed the presence of class 1 and 2 integrons, as indicated by the presence of the *Int*1 and *Int*2 genes from all the sampled hospices. The high occurrence of integrons in the housefly DNA from the hospices in this study may reflect that these class 1 and 2 integrons are widespread among the housefly bacterial communities. Integrons have been found in approximately 9% of the sequenced bacterial genomes, and the class 1 integron platform is the most ubiquitous and has been the most reported among clinical bacteria [57,58,59]. However, class 2 integrons are commonly reported in some species of Gram-negative organisms; they had significantly low occurrence and prevalence as compared with class 1 integrons [59]. The occurrence of class 1 and 2 integrons from the housefly bacterial communities was high in Lebone, followed by Sunflower and Sparrow hospices. The high occurrence of integrons in this hospice environment is of great concern because their antibiotic resistance determinants are known to be correlated with multidrug-resistant bacteria [28]. The hospices house immunocompromised and terminally ill patients who are very susceptible to infections caused by multidrug-resistant pathogens.

## 5. Conclusions

This study revealed a diverse composition of bacterial communities in the gut of houseflies and showed the presence of antibiotic resistance genes. Some of the characterized genera are pathogenic to humans and animals. The presence of bacterial species and the detection of antibiotic resistance genes from the housefly samples collected from the hospices indicates the possible health risk to patients in hospices and the surrounding communities, as the bacteria may be pathogenic and cause disease. Hence, it is imperative to keep high standards of hygiene, food preparation, safety, and control of houseflies in hospices. The findings obtained in this study open the door for future studies, particularly in determining the possibility of transmission of these microbes by houseflies and identifying candidate microbes that can be used to control the abundance of houseflies.

## Figures and Tables

**Table 1:** Summary of antibiotic resistance genes determined using PCR.

Resistance Gene Detected	Lebone Hospice n = 15 (%)	Sparrow Hospice n = 15 (%)	Sunflower Hospice n = 15 (%)	*p-*Value
Penicillin antibiotic resistance genes				0.231
*erm*B	2 (13.3%)	1 (6.6%)	0 (0%)	
*mec*A	0 (0%)	0 (0%)	0 (0%)	
*van*A	0 (0%)	0 (0%)	0 (0%)	
*β*-lactam antibiotic resistance genes				0.125
*blaCARB*	0 (0%)	0 (0%)	0 (0%)	
*blaTEM*	4 (26.7%)	0 (0%)	2 (13.3%)	
*blaSHV*	6 (40.0%)	0 (0%)	5 (33.3%)	
*amp*C	0 (0%)	0 (0%)	0 (0%)	
Tetracycline antibiotic resistance genes				0.122
*tet*A	9 (60%)	3 (20.0%)	5 (33.3%)	
*tet*W	0 (0%)	0 (0%)	0(0%)	
*tet*X	0 (0%)	0 (0%)	0 (0%)	
Integrons				0.783
*int*I	8 (53.3%)	6 (40.0%)	7 (46.7%)	
*int*II	9 (60.0%)	2 (13.3%)	8 (53.3%)	

n—number of antibiotic resistance genes; %—prevalence of antibiotic resistance genes.

## Data Availability

The datasets generated during and/or analyzed during the current study were deposited in the NCBI Sequence Read Archive (SRA): SRP356827 under the BioProject: PRJNA800672.

## Supplementary Materials

The following supporting information can be downloaded at: https://www.mdpi.com/article/10.3390/microorganisms11061440/s1, Figure S1: A stacked bar plot showing relative abundance of bacterial phyla detected from houseflies collected from three hospices in Bloemfontein and Johannesburg cities. Figure S2: A stacked bar plot showing relative abundance of bacterial class detected from houseflies collected from three hospices in Bloemfontein and Johannesburg cities and Figure S3: A stacked bar plot showing relative abundance of bacterial families detected from houseflies collected from three hospices in Bloemfontein and Johannesburg cities; Table S1: Primer sequences specific to different antimicrobial resistant determinants in housefly DNA extracts [28,60,61,62,63,64].
