# Supplementary material for: Bacterial Communities Associated with Houseflies (Musca domestica L.) Inhabiting Hospices in South Africa
Source: Microorganisms. 2023 May 30;11(6):1440. doi: 10.3390/microorganisms11061440 (PMC10304104; doi:10.3390/microorganisms11061440)
Supplement: Supplementary file 1 [file microorganisms-11-01440-s001.zip › microorganisms-2387207-supplementary.pdf]

microorganisms MDPI

Supplementary Materials

Characterization of Bacterial Communities and Antibiotic Resistant Genes from Houseflies Inhabiting Hospices in South Africa

Maropeng C. Monyama [1,2,*], Oriel M. Taioe [2,3], Jane S. Nkhebenyane [4], Deidre van Wyk [2], Tsepo Ramatla [2] and Oriel M. M. Thekisoe [2]

[1] Department of Life and Consumer Sciences, University of South Africa, Florida 1710, South Africa
[2] Unit for Environmental Sciences and Management, North-West University, Potchefstroom 2520, South Africa; taioem@arc.agric.za (O.M.T.); 20418876@nwu.ac.za (D.v.W.); ra21205450@gmail.com (T.R.); Oriel.Thekisoe@nwu.ac.za (O.M.M.T.)
[3] Epidemiology, Parasites and Vectors, Agricultural Research Council–Onderstepoort Veterinary Research, Pretoria 0110, South Africa
[4] Department of Life Sciences, Central University of Technology, Bloemfontein 9300, South Africa; snkheben@cut.ac.za
* Correspondence: monyamc@unisa.ac.za; Tel.: +27-18-299-2521

Citation: Lastname, F.; Lastname, F.; Lastname, F. Title. *Microorganisms* **2022**, *10*, x. https://doi.org/10.3390/xxxxx

Academic Editor: Firstname Lastname

Received: date
Accepted: date
Published: date

Publisher's Note: MDPI stays neutral with regard to jurisdictional claims in published maps and institutional affiliations.

Copyright: © 2022 by the authors. Submitted for possible open access publication under the terms and conditions of the Creative Commons Attribution (CC BY) license (https://creativecommons.org/licenses/by/4.0/).

Abstract: Houseflies are alleged reservoirs as well as vectors of human and animal pathogens, including bacteria, because they frequently have contact with animal excreta and decaying organic substances. The rapid adaptation process of ingested microbes in the insect gut may involve gene transfer, including antibiotic resistant determinants among different bacterial strains. As such, this study characterized bacterial communities harboured by houseflies collected from hospices using next-generation sequencing (NGS) platform and further seeked to detect antibiotic resistance traits by gene-specific PCR assays. Gene sequence analysis revealed that the most abundant phyla detected with variable abundance observed among all houseflies were Proteobacteria, followed by Firmicutes, Bacteroidetes, and Actinobacteria. Furthermore, NGS data revealed the presence of multiple bacterial genera, including *Providencia*, *Enterobacter*, *Dysgonomonas*, *Escherichia-Shigella*, *Klebsiella*, *Pseudomonas*, and *Streptococcus*, which are known to harbour potentially pathogenic species of animals and humans. Antibiotic resistant genes detected from the housefly DNA in this study included *erm*B, *tet*A, *blaSHV*, and *blaTEM*. Furthermore, these genes are associated with resistance to erythromycin, tetracycline, and beta-lactams antibiotics respectively. The presence of bacterial pathogens and detection of antibiotic resistant genes from houseflies collected from the hospices indicates the possible health risk to patients in hospices and the surrounding community. Therefore, it is imperative to keep high standards of hygiene, food preparation, safety, and control of houseflies in hospices.

Keywords: bacterial communities; 16*S Rrna*; houseflies, resistance genes

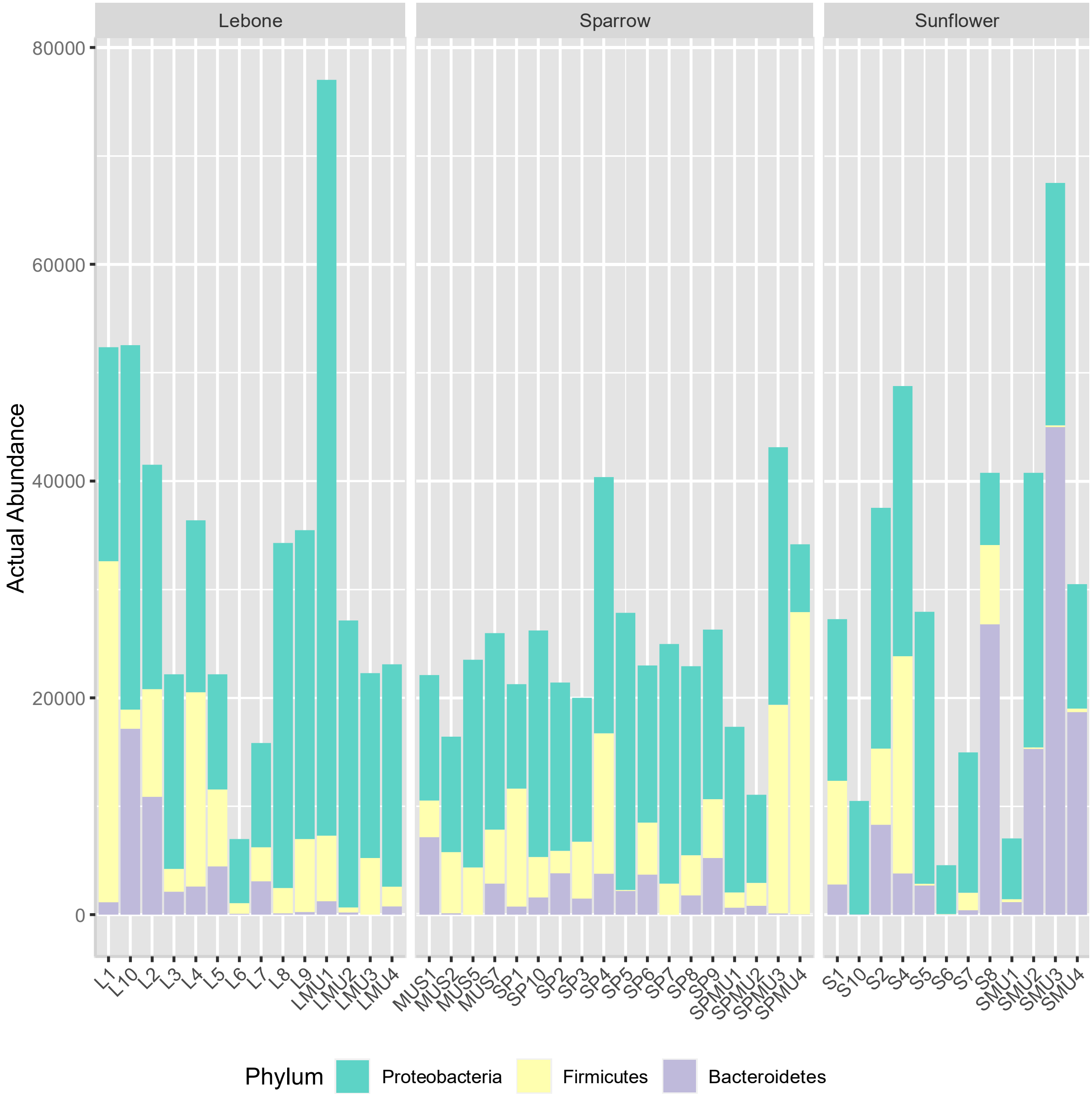

Figure S1. A stacked bar plot showing relative abundance of bacterial phyla detected from houseflies collected from three hospices in Bloemfontein and Johannesburg cities.

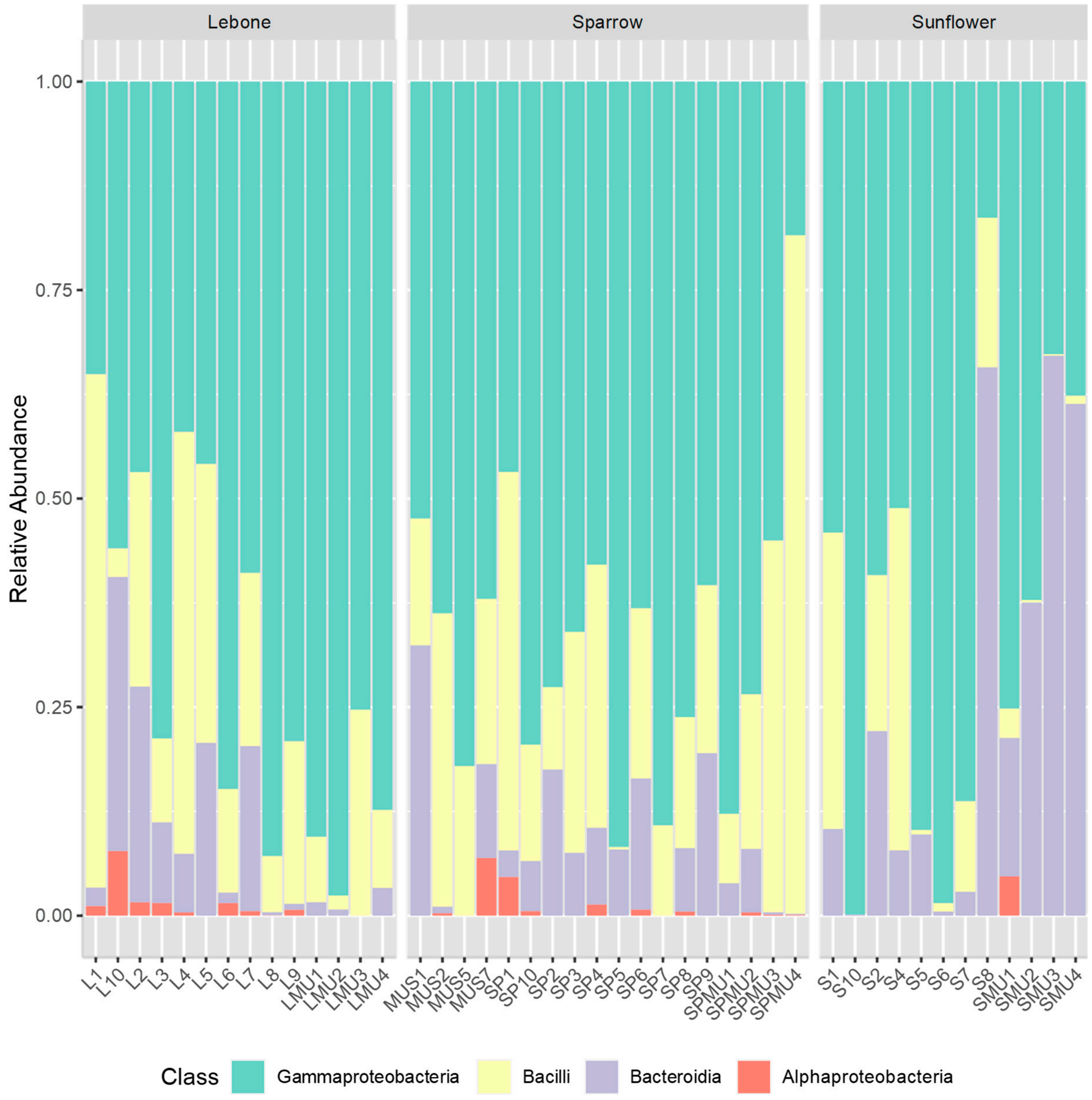

Figure S2. A stacked bar plot showing relative abundance of bacterial class detected from houseflies collected from three hospices in Bloemfontein and Johannesburg cities.

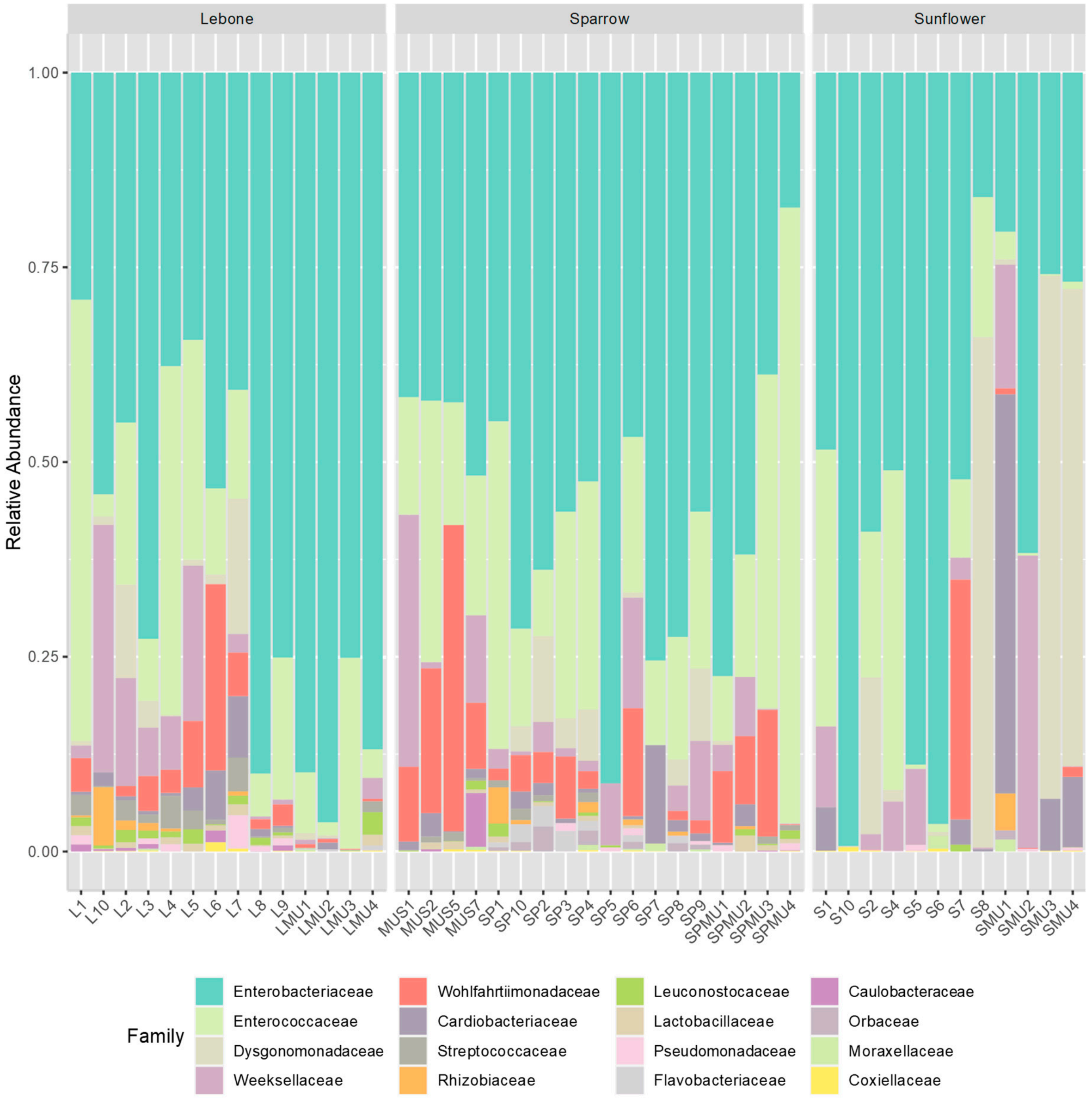

Figure S3. A stacked bar plot showing relative abundance of bacterial families detected from houseflies collected from three hospices in Bloemfontein and Johannesburg cities.

Table S1. Primer sequences specific to different antimicrobial resistant determinants in housefly DNA extracts

Resistant Gene Detected	Primer Direction	Sequence (5'–3')	Annealing Temperature (°C)	Amplicon Size (bp)	References
Macrolide antibiotic-resistant genes					
*erm*B	F	GCATTTAACGACGAAACTGGCT	61	573	[28]
	R	GACAATACTTGCTCATAAGTAATGGT			
*mec*A	F	TAATAGTTGTAGTTGTCGGGTTTG	61	733	[28]
	R	TAACCTAATAGATGTGAAGTCGCT			
*van*A	F	GAAATCAACCATGTTGATGTAGCA	61	572	[28]
	R	TTTGCCGTTTCCTGTATCCGT			
β-lactam antibiotic-resistant genes					
CARB	F	CAAGTACTTTYAAAACAATAGC	46	534	[28]
	R	GCTGTAATACTCCKAGCAC			
TEM	F	AAAGATGCTGAAGATCA TTTGG-	44	425	[28]
	R	TATGGCTTCATTC			
	F	GCGAAAGCCAGCTGTCGGGC	62	304	[28]
SHV	R	GATTGGCGGCGCTGTTATCGC			
Tetracycline antibiotic-resistant genes					
*tet*A	F	GCGCTNTATGCGTTGATGCA	62	387	[28]
	R	ACAGCCCGTCAGGAAATT			
*tet*W	F	GAGAGCCTGCTATATGCCAGC		168	[28]
	R	GGGCGTATCCACAATGTTAAC	64		
*tet*X	F	CCGACACGGAAGTTGAAGAA CCTT-		468	[28]
	R	GGTGAGATGCCATTAGC	60		
Integrons					
*int*I	F	GATGCCTGCTTGTTCTAC	55	558	[60]
	R	GCCTTGCTGTTCTTCTAC			
*int*II	F	GTAGCAAACGAGTGACGAAATG	62	788	[61]
	R	CACGGATATGCGACAAAAAGGT			
16S *rRNA*		16S *rRNA*			
	F	ATGGCTGTCGTCAGCT		440	[62]
	R	ACGGGCGGTGTGTAC	-		

Virulence genes					
invA	invA 139	GTGAAATTATCGCCACGTTCGGGCAA	53	284	[63]
	invA 141	TCATCGCACCGTCAAAGGAACC			
sefA	S1	GCCGTACACGAGCTTATAGA	53	250	[63]
	S4	ACCTACAGGGGCACAATAAC			
fliC	Fli15	CGGTGTTGCCCAGGTTGGTAAT	53	620	[63]
	Typ04	ACTGGTAAAGATGGCT			
uge	F	TCT TCA CGC CTT CCT TCA CT	54	534	[64]
	R	GAT CAT CCG GTC TCC CTG TA			
magA	F	GGT GCT CTT TAC ATC ATT GC	59	1280	[64]
	R	GCA ATG GCC ATT TGC GTT AG			
kfu	F	GAA GTG ACG CTG TTT CTG GC	54	797	[64]
	R	TTT CGT GTG GCC AGT GAC TC			
